# Looking for interaction: quantitative measurement of research utilization by Dutch local health officials

**DOI:** 10.1186/1478-4505-10-9

**Published:** 2012-03-13

**Authors:** Joyce de Goede, Marja JH van Bon-Martens, Kim Putters, Hans AM van Oers

**Affiliations:** 1 Academic Collaborative Centre of Public Health Brabant, Tilburg University, Tilburg, the Netherlands; 2 Regional Public Health Service West-Brabant, Breda, the Netherlands; 3 Regional Public Health Service Hart voor Brabant, 's-Hertogenbosch, the Netherlands; 4 Institute of Health Policy and Management, Erasmus University, Rotterdam, the Netherlands; 5 Department of Public Health Status and Forecasts, National Institute of Public Health and the Environment, Bilthoven, the Netherlands

## Abstract

**Background:**

In the Netherlands, local authorities are required by law to develop local health memoranda, based on epidemiological analyses. The purpose of this study was to assess the actual use of these epidemiological reports by municipal health officials and associated factors that affect this use.

**Method:**

Based on a conceptual framework, we designed a questionnaire in which we operationalized instrumental, conceptual, and symbolic use, the interaction between researchers and local health officials, and four clusters of barriers in this interaction process. We conducted an internet survey among 155 Dutch local health officials representing 35% of all Dutch municipalities. By means of multiple regression analyses, we gained insight into the related factors for each of the three types of research utilization.

**Results:**

The results show that local health officials use epidemiological research more often in a conceptual than an instrumental or symbolic way. This can be explained by the complexity of the local policy process which is often linked to policies in other areas, and the various policy actors involved. Conceptual use was statistically associated with a presentation given by the epidemiologist during the policy process, the presence of obstructions regarding the report's accessibility, and the local official's personal belief systems and interests originating from different professional values and responsibilities. Instrumental and symbolic use increased with the involvement of local officials in the research process.

**Conclusions:**

The results of this study provide a partial solution to understanding and influencing research utilization. The quantitative approach underpins earlier qualitative findings on this topic. The outcomes suggest that RPHS epidemiologists can use different strategies to improve research utilization. 'Blurring the boundaries', and the enhancement of interfaces between epidemiologists and local health officials, like direct interactions into each other's work processes, is expected to create better possibilities for optimizing research use.

## Background

In recent years, research utilization has been a growing scientific field. As Nutley et al. (2007) stated: "Research use is a complex and multifaceted process, and the use of research often means different things for different people" [1]. In public health discourse, "use" is mainly acknowledged if research causes a change of policy. Research use in the sense of increasing the general body of knowledge is not taken into account and research use as ammunition during policy discussions, is often regarded as 'mis'use [2]. Many health professionals perceive research utilization as important for improving health at population level, related to the increasing importance of the concept of 'evidence based policy' (EBP). Thereby it is assumed that EBP will offer the best possibilities for improving population health. EBP means the conscious, explicit, and judicious use of the best available evidence [3] during the policy process. The term 'evidence-informed' can also be used, to stress the role of evidence and the ambition to improve the extent to which research evidence leads to informed decisions [4].

In the Netherlands, the Dutch Public Health Act builds on the concept of EBP, stating that local authorities are required to establish a local public health memorandum every four years on the basis of (local) epidemiological research. The regional or local epidemiological reports that are produced for this purpose are mainly based on health surveys, and have a descriptive nature. For example, the reports describe the frequency of different health measurements such as the occurrence of diseases or quality of life. They also describe the occurrences of determinants of health such as lifestyle and social and environmental factors. The epidemiological research data is provided by 28 Regional Public Health Services (RPHS) serving all 430 Dutch municipalities. However, it is yet not known whether and how the epidemiological reports are used by the local health officials who receive them.

The aim of this paper is to determine how and to what extent the RPHS epidemiological research is used by local health officials, and to identify the factors that influence this use. Qualitative studies are valuable for identifying the mechanisms of research use, but to gain insight into the nature of utilization and finding influencing factors, a quantitative approach is more suitable. Earlier municipal case studies [5] have shown that the local health officials fulfill a key role in the distribution of epidemiological information and knowledge during the local health policy development process, so a survey was carried out among these local health officials. These local health officials are professional practitioners and work under the supervision of elected administrators. They are responsible for the development of the local health policy memoranda and in many cases also for implementing the policy.

In this article we start with the explanation of a conceptual framework as a base for this study. In the methodological section we describe how this framework is operationalized into survey questions. In the following result section we will present the descriptive statistics and the linear regression models. We close the article with an extensive discussion concerning the results, methods and the conceptual framework and end with final conclusions.

### Development of a conceptual framework

**Figure 1:**
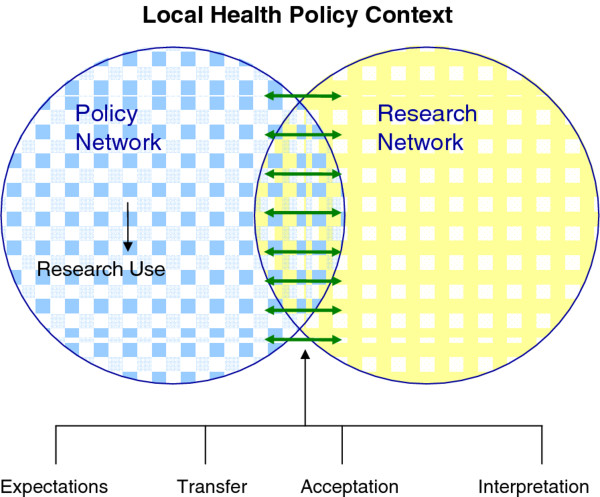
**Conceptual framework for analyzing the use of epidemiological research for local health policy**.

There is an extensive body of international literature on research utilization which is still growing. In an earlier review published in this journal, we developed a conceptual framework on research utilization in this specific Dutch context [6]. This framework is shown in Figure 1. First, we state that an epidemiological research report is produced in a network of researchers. Second, the report is received by several policymakers who all are related to one another in a policy network. In the theoretical literature on research utilization, interaction is seen as an important precondition for translating research findings into policy [7-14]. Interaction can be defined as the reciprocal actions of two or more people who work together, negotiate on opinions, values and norms and find consensus. In practice this means either that policymakers are directly or indirectly involved in the research process or that researchers are involved in the policy process. In our conceptual framework, this interaction, and consequently research use, can be obstructed by several barriers, which we have divided into four domains. The Expectation domain addresses the issue of awareness among researchers and policymakers of each other's 'niches' [15], containing barriers that can be acted upon during the preparation phase of research. The *Transfer *domain refers to the publication phase of the research cycle, addressing research communication. In another case study conducted in the Netherlands [16], it became clear that media attention can be very influential. Therefore we added this item to the conceptual framework in the transfer domain. Two other domains, *Acceptance *and *Interpretation*, both contain barriers relating to the individual attributes of the officials. *Acceptance *barriers refer to the personal perception of the validity of the research outcome, (not to be confused with scientific validity). *Interpretation *barriers refer to the meaning each person gives to research outcomes. In the conceptual framework all associated factors are described separately however in practice it will be possible that these factors themselves are interrelated [6].

There are various quantitative measurements for research utilization. Many quantitative studies have used the ladder of research utilization of Knott and Wildavsky, a measure of main outcome [8,17-19]. However, as a result of our municipal case studies [5], we became more interested in the different ways of usage because we noted that the same research can be applied in several ways. Therefore we followed Amara et al., and distinguished three types of use for individual policymakers: instrumental, conceptual, and symbolic [20]. Instrumental use means that the research is acted upon in specific and direct ways, for example to solve a problem at hand. Conceptual use means that the research improves the understanding of the subject matter and related problems, and refers to a more general and indirect form of enlightenment. Symbolic use means that, 1) research is used to justify a position or course of action for other reasons such as someone's own interests that have nothing to do with the research findings (political use), or 2) the fact that research is being done is exploited to justify inaction on other fronts (tactical use).

In earlier municipal case studies [5] it was shown that epidemiological research utilization of the local health officials also depended on the characteristics of the policy memoranda and how the policy process was organized. We believe these associated factors belong to the setting of the policy network in our conceptual framework, and it is therefore important that they are taken into account in the current study.

## Method

### Data collection

In the absence of a national list of Dutch local health officials, we approached all 28 Dutch RPHSs, asking them to cooperate with our study by providing us with the names and phone numbers of the municipal public health officials in their working area. 20 RPHSs cooperated, covering 339 municipalities. Reasons for the RPHSs not to cooperate were lack of time, other priorities, different timing of the development of the local health memoranda, and participation in other research projects regarding public health policy. Four research assistants approached the 339 local health officials by phone between November 2008 and April 2009, and asked them to participate. Those participating were asked to provide some background information such as the number of years working on this policy issue in this municipality, what other policy issues they have in their portfolio (e.g. social welfare, youth, or the elderly), and their education and research experience. Subsequently we asked for their email addresses, and sent them a protected link to an internet questionnaire. In December 2008, all approached officials received a reminder in the form of a digital Christmas card, and in February 2009 the respondents who had not yet filled in the questionnaire received again a reminder by email.

### Measurement of epidemiological research use

In an earlier study Amara et al. only used one question for each of the concepts of instrumental, conceptual and symbolic use. Given the specific Dutch context we designed multiple questions for each concept, since the concepts can have several meanings [5]. This was also suggested by Ouimet et al. [16], who pointed out that, in order to obtain more understanding of research utilization, more precise questions are needed. The questions we developed initially were pre-tested by, and discussed with, ten practitioners in the public health field (three RPHS epidemiologists, four RPHS local health policy advisors, and three local health officials).

The concept of instrumental use, referring to direct and concrete action due to the specific research results, was measured using two questions that asked whether research results had led to (1) new direct policy actions; and (2) the termination of one or more existing policy activities. Conceptual use was measured using three questions that asked whether the research results had led to (1) a better understanding of the occurrence and causes of health problems within the RPHS region; (2) a better understanding of the causes and occurrence of health problems within the municipality; and (3) new long-term ideas for projects or policies within the municipality or RPHS region. Finally, symbolic use was measured using two questions that asked if, due to the research, the officials were able to (1) question existing policies and decisions; and (2) put personal ideas on the policy agenda. All questions had a 5-point Likert-type response scale ranging from (1) not applicable in my situation; (2) minimally applicable in my situation; (3) moderately applicable in my situation; (4) applicable in my situation; (5) strongly applicable in my situation.

### Measurement of the associated factors

All independent variables are shown in Tables 1, 2, 3 and 4.

**Table 1:** Associated factors of research utilization for policy and epidemiological reports (n = 155)

*variables*	*response categories*	*%*
**Policy memorandum**		

Composition of the memorandum	Memorandum specific for my own municipality	61,3
	
	Memorandum composed with other municipalities with a local section	38,7

Type of memorandum	Memorandum solely about public health	78,7
	
	Memorandum combines public health issues with other policy issues such as welfare	21,3

**Policy process**		

The initiative to start the memorandum	Local administrators	49,7
	
	City council	10,3
	
	Local official	24,5
	
	RPHS	10,3

Receive support on the decision making process from the Registry office	Yes	14,8
	
	No	85,2

Council members are involved during policy preparation before they had to make a decision	Yes	31,6
	
	No	68,4

One or more welfare and health care organizations are involved during policy preparation	Yes	81,3
	
	No	18,7

One or more organizations of client representatives are involved during policy preparation	Yes	82,6
	
	No	17,4

One or more local officials working on other policy issues are involved during policy preparation	Yes	95,5
	
	No	4,5

**Epidemiological report**		

Geographical area of the epidemiological research	Local level only	12,9
	
	Regional level only	10,3
	
	Local as well regional level	71,6

		median: 18

Number of public health topics that can be mentioned in the epidemiological reports

**Table 2:** Associated Factors of research utilization for interaction (n = 155)

*variables*	*response categories*	*%*
**Interaction**		

Involved in the research process at any given moment	Yes	38,1
	
	No	61,9

Involvement in policy process by RPHS	RPHS professionals including epidemiologists	52,3
	
	RPHS professionals excluding epidemiologists	40,0
	
	No RPHS professionals involved	7,7

Epidemiological health reports presented during policy development	Yes	44,5
	
	No	55,5

**Table 3:** Associated factors of research utilization for barriers (n = 155)

*%*	Totally agree	Agree	Neither agree nor disagree	Disagree	Totally disagree
Relevant for local health policy (expectations)	5,2	47,1	41,3	5,2	1,3

Sufficiently related to other policy domains (expectations)	17,4	28,4	33,5	15,5	5,2

Content is sufficiently current (expectations)	2,6	34,8	41,9	17,4	3,2

Report is presented to me on time (expectations)	5,2	37,4	50,3	5,8	1,3

Satisfied with the structure of the report (transfer)	32,3	38,1	22,6	5,2	1,9

Report was easy to understand (transfer)	46,5	34,2	14,8	3,9	0,6

Sufficient regional information (transfer)	27,1	32,9	14,2	18,7	7,1

Sufficient local information (transfer)	43,2	39,4	15,5	1,3	0,6

RPHS is perceived as a credible source (acceptance)	51,6	32,9	11,6	1,3	2,6

RPHS made the basis of the epidemiological finding clear (acceptance)	40,6	38,1	16,8	1,9	2,6

Suited/Fitted well with personal belief system regarding local health policy (acceptance)	25,2	34,2	32,9	6,5	1,3

Suited the contemporary political vision on public health within the municipality (interpretation)	22,6	30,3	37,4	7,1	2,6

**Table 4:** Additional associated factor of research utilization for media n = 155)

*variables*	*response categories*	*%*
Media attention (transfer)	Mainly positive publications	16,1
	
	Mainly negative publications	1,3
	
	Mainly neutral publications	16,8
	
	Variable publications	8,4
	
	No publications	9,0
	
	Not familiar with any publications	48,4

Additional research information from other sources	Yes	67,1
	
	No	32,9

For the policy context (as part of the policy network in the conceptual framework), we first defined six categories of background variables: the size of the municipality, the urban nature of the municipality, the number of years worked as a local health official in this municipality, whether the local health official had to consider other policy issues in his daily job besides public health (dichotomous), the educational level of the official, and his experience in conducting research. Secondly, we defined two categories of variables relating to the public health memorandum: the composition of the memorandum (memoranda solely for their own municipality or written together with other municipalities), and the type of the memorandum (memorandum solely about public health or combined with other policy domains). Thirdly, we defined six questions concerning the decision-making process: (1) Who took the initiative to start the memorandum (categorical)?; (2) Did they receive support in the decision making process from the municipal Registry office (dichotomous)?; (3-6) four questions asking who took part in the policy preparations (city council members, local health care providers, local client representatives, colleague officials from related policy domains such as welfare or youth - all dichotomous). For the content of the epidemiological research information, we asked which geographical area was covered, and, from a list of 22 topics, which public health topics were described, (for example, death rates, indicators for quality of life, presence of chronic diseases, lifestyle-related risk factors, social risk factors). The frequencies of all these associated factors are shown in Table 1.

For the measurement of the actual interaction between the RPHS epidemiologists and the local health officials, we used three questions based on the 'burring the boundaries' model of de Leeuw et al. [13]. In this model, interaction is defined as actions undertaken by policy makers during the research process, and conversely by researchers during the policy process, in order to influence these processes. Therefore, we asked whether (1) the local officials were involved in the research process at any given moment (dichotomous); and (2) whether RPHS officials were involved in the policy process. Three answers were possible for this question: epidemiologists (with or without other RPHS professionals), only other RPHS professionals, or the RPHS professionals were not involved at all. Additionally we asked if an oral presentation about the epidemiological research had been given by the RPHS during policy preparation (Table 2).

For the measurement of possible barriers to research utilization within the interaction, we operationalized the barriers (in each case, noting which of the four domains is applicable) (Table 3). Questions for measuring barriers in the expectation domain asked whether the epidemiological research was (1) considered relevant for local health policy; (2) sufficiently related to other policy domains; (3) current; and (4) on time. Questions for measuring barriers in the transfer domain asked whether (1) the respondent was satisfied with the structure of the report; (2) the respondent was satisfied with the accessibility of the report regarding intelligibility; (3) the respondent thought the report contained enough regional information; (4) the respondent thought the report contained enough local information; (5) there had been media attention due to the epidemiological report (Table 4); and (6) the respondent had additional research information from other sources(Table 4). Questions for measuring barriers in the acceptance domain asked whether (1) the respondent trusted the RPHS as a credible source for epidemiological research; (2) the RPHS made clear what the epidemiological research was based on; and (3) the epidemiological report suited the respondent's personal belief system regarding local health policy. Finally, questions for measuring the barriers in the interpretation domain asked whether the content of the epidemiological report was in line with the current political vision on public health within the municipality.

All barrier questions, except for media and the use of other research sources, had the following 5-point Likert-type response scale: totally agree, agree, neither agree nor disagree, disagree, totally disagree.

### Statistical analysis

In social science literature there is an ongoing debate [21] about whether or not it is legitimate to use Likert scale data in parametric statistical procedures that require interval data, such as Linear Regression. In our case the underlying concept from the Likert items can be regarded from not applicable to very strong applicable as interval-level data and therefore we used linear multiple regression analyses to determine which independent variables were associated with each type of use of epidemiological research (instrumental, conceptual, symbolic) [22]. For each linear regression model, we first constructed a scale for research use if appropriate, based on all the responses to the questions involved. Secondly, we made a selection of independent variables to be included in the model, based on their univariate associations with research use and their mutual associations. All analyses were carried out with SPSS Statistics 17.0.

#### Construction of scales for research use

For each type of research use, the internal reliability coefficient of the corresponding questions was calculated, using Cronbach's Alpha [23]. When the value of Cronbach's alpha exceeded a score of 0.60, we concluded that the internal consistency of the questions was reliable, and combined the responses of the questions into one sum score [24]. If Cronbach's alpha was 0.60 or lower, we chose the one question that, in our opinion, best covered the concept for the Dutch situation.

#### Selection of associated variables

Since the research population is rather small, only a limited number of independent variables can be included in the regression models [23]. For each regression model, the unconditional relations of the independent variables with research use were tested using one-way Anova. The independent variables with a significant test result (p < 0.05) were further tested for their mutual correlations in order to avoid multicollinearity. Depending on the nature of the variables (categorical or continuous) we used a Chi-square test, a one-way Anova, or a Pearson correlations coefficient. Correlated variables (based on p < 0.05) were then combined into one variable. If this was necessary we describe the development of the new combined variable in the result section. No correlations between continuous and categorical variables occurred. Additionally, dummy coding was used to convert the categorical variables into dichotomous dummy variables.

## Results

### Response

In total, 284 of the 339 eligible local health officials consented to collaborate in the study. Officials who did not want to participate, were either not interested, had no time, or sometimes indicated a poor relationship with their RPHS. After the follow-up email invitation, 224 local health officials started the internet questionnaire, eventually leading to 173 completed questionnaires. This is a response of 51% and covers 39% of all Dutch municipalities.

We then excluded 18 respondents who acknowledged that, although they were involved in the policy process and could reproduce information on this, they did not know the epidemiological reports, and therefore were not able to give their opinions on interaction and barriers to research use. Mostly, these officials had been working in their present function for less than three months. As a result of their exclusion, 155 questionnaires were included in our analysis, covering 35% of all Dutch municipalities.

Population size is a factor that influences the development of local health policy, and is related to the capacity of civil servants assigned [23]. If we compare the distribution of population size of the municipalities in the study with all Dutch municipalities we see that there were only minor differences in the distribution of population size. Municipalities in our study were slightly more often medium sized, and less often small.

### Descriptive statistics of the associated factors

Tables 1 to 4 show the descriptive results of the all associated variables. The municipalities in which the 155 respondents worked varied in population size and urban nature. The experience of the respondents in their current position was diverse. Most of them (34%) had had five to ten years of experience. Almost all respondents had served in a variety of policy areas besides public health. Social services, youth, and the elderly were mentioned most frequently. Most of the respondents held a Bachelors degree (41%) or a Masters degree (47%). Furthermore, approximately one third of the respondents had no personal experience with research. The others had experience of qualitative research, quantitative research, or both.

### The use of epidemiological research in local health policy development

Table 5 shows that conceptual use was the most common type of research use in the development of local health policy. The questions for conceptual use had the highest mean scores (2.80, 2.77, and 2.77). Instrumental use was the least common type of research use.

**Table 5:** Frequency distribution of instrumental, conceptual, and symbolic use by Dutch public health officials

		not applicable in my situation	minimal application in my situation	moderately applicable in my situation	applicable in my situation	strongly applicable in my situation	Total	Mean	SD
Instrumental use	I have recently started new concrete policy activities within my municipality	43%	19%	21%	11%	6%	100%	2,17	1,25
	
	I have stopped certain policy activities within my municipality	91%	3%	8%	X	X	100%	1,15	0,49

Conceptual use	I have a better understanding of the health problems and their causes within the RPHS region	24%	16%	22%	31%	7%	100%	2,8	1,3
	
	I have a better understanding of the health problems and their causes within my municipality	22%	18%	28%	26%	6%	100%	2,77	1,23
	
	I have developed new ideas for the long term for projects or policies within my municipality or in collaboration with other organisations	22%	17%	28%	26%	6%	100%	2,77	1,24
	
	Sum score for conceptual use							9.0	2.99

Symbolic use	I have been able to discuss existing policies and activities within my municipality	46%	18%	22%	10%	5%	100%	2,1	1,22
	
	I have been able to place my personal ideas and preferences on the policy agenda	38%	19%	21%	17%	6%	100%	2,34	1,29
	
	Sum score for symbolic use							4.7	2.17

X: this response category was not mentioned

For instrumental use, Cronbach's alpha for the two sub questions was too low (α = 0.230) to combine them into one variable. For further regression analysis, we therefore decided to use the first question ("I have recently started new concrete policy activities within my municipality") as dependent variable, because, in our opinion, it covered the concept of instrumental use sufficiently well, and had enough respondents in each category.

The internal reliability of the three sub-questions for conceptual use was high enough (Cronbach's α = 0.841) to sum their scores into one score. The mean sum score for conceptual use was 9.0 (SD = 2.99).

Regarding symbolic use, the value of the internal reliability was also sufficient to combine the two sub-questions (Cronbach's α = 0.66). The mean sum score for symbolic use was 4.7 (SD = 2.17).

### Results of the linear regression models

Table 6 shows the results of the linear regression models for each of three types of research use.

**Table 6:** Regression models on instrumental, conceptual, and symbolic use by Dutch public health officials

Typology of research use	N = 155	B	β	t	p
Instrumental use	(constant)	2,245		4,391	0,000

	Personal experience with research				

	no personal experience with research (ref category)				

	mainly experience with qualitative research	0,405	0,131	1,534	0,127

	mainly experience with quantitative research	-0,530	-0,139	-1,705	0,090

	experience with both types of research	0,376	0,140	1,663	0,099

	Involvement of the local health official in the research process				

	No local officials involved in the research process (ref category)				

	Local officials involved in the research process at any given moment	0,626	0,242	3,167	0,002*

	Involvement of the RPHS in the policy process				

	No involvement of the RPHS with the policy process (ref category)				

	RPHS professionals including epidemiologists involved in policy process	-0,158	-0,063	-0,435	0,664

	RPHS professionals including epidemiologists involved in policy process	-0,569	-0,223	-1,562	0,121

	Media attention				

	no media publications (ref category)				

	mainly positive media publications	-0,631	-0,185	-1,630	0,105

	mainly negative media publications	0,962	0,087	1,064	0,289

	mainly neutral media publications	-0,537	-0,160	-1,402	0,163

	variable media publications	-0,571	-0,126	-1,276	0,204

	no familiarity with any media publications	-0,817	-0,326	-2,438	0,016*

Conceptual use	(constant)	10,554		7,853	0,000

	Involvement of the local health official in the research process				

	No local officials involved in the research process (ref category)				

	Local officials Involved in the research process at any given moment	0,534	0,087	1,206	0,230

	Involvement of the RPHS in the policy process				

	no involvement of RPHS and no presentation was given (ref category)				

	epidemiologist involved in the policy process and gave a presentation	2,839	0,422	3,266	0,001*

	epidemiologist involved in the policy process but did not give a presentation	1,087	0,158	1,247	0,214

	other RPHS professionals were involved and gave a presentation	1,612	0,198	1,731	0,086

	other RPHS professionals were involved, and no presentation was given	1,004	0,143	1,146	0,254

	Presence of barriers	-0,152	-0,350	-4,815	0,000*

Symbolic use	(constant)	3,545		6,849	0,000

	Involvement of the local health official in the research process				

	No local officials involved in the research process (ref category)				

	Local officials Involved in the research process at any given moment	0,871	0,354	2,464	0,015*

*significant with p < 0,05

Four independent variables were significantly and unconditionally related to *instrumental use*, as tested with one-way Anova: 1) experience with research (F = 3.70, df = 3, p < 0.01,), 2) involvement of the local official with the research process (F = 14.04, df = 1, p < 0.01), 3) involvement of the RPHS with the policy process (F = 3.37, df = 2, p < 0.05), and 4) media attention (F = 2.47, df = 5, p < 0.05). Chi-square tests between these associated factors showed that they were not interrelated. Therefore, all four factors were included in a linear regression analysis. Table 6 presents the resulting model, which explained a significant amount of variance in instrumental research use (adjusted R^2 ^= 0.17, F = 3.93, p < 0.01). The model shows that the involvement of local officials was significantly related to more instrumental use, whereas unawareness of local officials of a media publication about the epidemiological report was significantly related to less instrumental use.

Fourteen associated factors were significantly related to *conceptual use*: all three actual interaction variables and eleven of the thirteen barrier variables. Only media attention and satisfaction with the local information were not statistically significantly related to conceptual use. The tests for mutual correlation showed that involvement of the RPHS in the policy process was related to an oral presentation on the epidemiological report. Moreover, all barrier variables were interrelated (^Q3^Additional file 1: Appendix 1). For the linear regression model, we created a new combined categorical variable for actual interaction, with five response categories: 1) an epidemiologist was involved in the policy process and gave a presentation, 2) an epidemiologist was involved in the policy process but did not give a presentation, 3) other RPHS professionals were involved and gave a presentation, 4) other RPHS professionals were involved, but no presentation was given, 5) the RPHS was not involved in the policy process at all (see ^Q4^Additional file 2: Appendix 2 and Table 6). Based on a reliability calculation of the eleven selected barrier variables (Cronbach's alpha = 0.88), we concluded that these questions had sufficient internal consistency to combine them. The scores of all eleven questions were summed up into one score for barriers, which varied between 11 (no barriers present) and 55 (all barriers present), and had a mean of 24.96 (SD 6.87). Table 6 presents the resulting model for conceptual use, which significantly explained the variance (Adjusted R^2 ^= 0.227, F = 8.541, p < 0, 01). There was a relation between, on the one hand, the involvement of, and a presentation by, an epidemiologist in the policy process and, on the other hand, a higher sum score for conceptual use of the research. However, as mentioned by the local officials, conceptual use decreased with a higher sum score for barriers to interaction.

As for *symbolic use*, only one associated factor was significantly related: involvement of the local official during the research process (F = 6.071, df = 1, p < 0, 05). Table 6 presents the resulting model for instrumental use, which had a low explanation of the variation (Adjusted R^2 ^= 0.032, F = 6.071, p = 0.015). It showed that interaction during the research process increased symbolic use, as mentioned by the local health officials.

## Discussion

The aim of this paper was to quantify the nature and extent of epidemiological research use in the Netherlands during the development of municipal public health policy, and the factors that determine this use. We conducted a survey among local health officials because, in earlier case studies [5], it was shown that they played a key role in the development of local health policy.

This study provides very specific insight into this specific research population of Dutch local health officials. Conceptual use was more common than instrumental and symbolic use. This means that the knowledge and insights of the epidemiological reports are not translated into concrete actions nor are they used in policy debates. The greater amount of conceptual use was also found by Amara et al., although they conducted their survey among professionals and managers in government agencies in Canada [19]. In our study, the level of conceptual use, as well as that of instrumental and symbolic use, is higher than in the study of Amara et al. [20]. One explanation for this is the difference in research population in a specific Dutch local policy area. The study population of Amara et al. was more diverse and contained also managerial, regional and national officials [18]. Another explanation could be that we operationalized the concept of research utilization by using different and multiple questions. If we consider the higher amount of conceptual use from the local Dutch policy making context this can be explained by the process of the policy process and diversity of other policy actors playing in the field of local health policy. The local official has to take account of the knowledge, opinions, and interests of other actors, and is therefore not able to directly transform the recommendations of the epidemiological report into action. The importance of the health frames of policy actors and their belief systems and interests determine the outcomes of the health policy process [5,25]. It remains the questions to what extent it is possible for researchers to take all these different perspectives into account during the research process. Giving the complexity of the policy process it is debatable whether evidence based policy and instrumental use of epidemiological knowledge are actually the proper goals to strive for. It would probably be better to emphasize on conceptual use and aim for higher awareness and better understanding of the provided epidemiological research knowledge so we can speak about evidence informed policy. Ultimately if conceptual use of research is high during the policy process and applies for multiple policy actors, eventually this can lead to more instrumental use.

Instrumental use can be explained by preliminary interaction between researcher and policy makers during the research process, in other words, the involvement of local officials during the design of the research and during the publication phase. However, it is harder to understand a lack of awareness by local officials of a media publication on the epidemiological report as a factor for less instrumental use. Because of the cross-sectional design of this study, this association could be the other way round. If a person does not see many possibilities of using an epidemiological report instrumentally ("I do not have to do anything with it"), he might have less interest in media attention.

Our results showed that the presentation of the health report was associated with greater conceptual use. This would imply that more value is given to the epidemiological knowledge when presented by an epidemiologist than when presented by another employee of the RPHS. This can probably be explained by the perceived authority of an epidemiologist. In the conceptual framework, we distinguish four domains of barriers to research use. This study shows that most of these barriers are interrelated, so we are not able to assess which barriers are more important.

For symbolic use, the only factor was the preliminary interaction, which can explain only limited variance. This means that there must be other factors than those included in our study that explain variance. On the one hand this outcome can be explained by the fact that, during the policy process, local officials do not always take part in the policy discussion but function as process manager. It can be expected that if we had asked administrators, politicians, or client representatives, the symbolic use would have been greater. On the other hand, we may have missed other factors such as the political composition of the municipal council or the political background of the local administrator.

The response to the study was 51%, covering 35% of all Dutch municipalities. A study by van Dijk [26] showed that the development of local health policy differs between municipalities of different size and urban nature. This is related to the time that the local official has available for the specific subject. However we showed that the distribution of the sizes of the municipalities in the research population corresponds with the national distribution in the Netherlands. Therefore we believe the response is sufficient to represent all Dutch municipalities.

### Reflections on methodology

There are some methodological limitations of this study that we have to discuss. We recruited the local health official by means of the RPHS. This was because there was no central list of local health officials in the Netherlands, and people regularly change jobs in the policy domain. We believed that the RPHS was the best possible source for the most recent list. However, this use of the RPHS was a potential cause of bias, because those organizations that were willing to cooperate with our study possibly put more emphasis on research utilization. Another form of selection bias could have occurred because officials with a negative attitude towards the RPHS might have been less willing to cooperate in our study. Both types of selection bias can cause an overestimation of research utilization by the group of local health officials. This could have the consequence that the regression models we produced are valid only if there is a neutral or positive relationship between the local health officials and the employees of the RPHS.

We chose a specific analysis strategy for the construction of the regression models. This decision was made because of two problems with the data. First is that we had a relatively small research population in comparison with the amount (more than twenty) explaining variables. Second, as shown in Additional file 1: Appendix 1, we were faced with the situation that the explaining, "independent" variables were strongly associated. This strategy could have caused us to miss variables not directly related to research use but that have an indirect influence by interacting with other variables. It is possible that this is related to the small magnitude of the found associations (Table 6) but that could also be caused by the relatively small amount of respondents (n = 155). However, the findings of this study must be must be confirmed in future research.

As we mentioned before in the methodology section we choose for linear multiple regression analyses to determine which independent variables were associated with each type of use of epidemiological research (instrumental, conceptual, symbolic). In the case of conceptual use this can be debatable [27] and one can question whether or not an ordinal regression analysis should not be more applicable to perform. To be sure we performed an ordinal regression and the outcomes can be found in ^Q5^Additional file 3: Appendix 3. Here we see that the same independent variables, namely preliminary interaction between researcher and policy makers during the research process and awareness about media attention, are significant.

The ladder of research utilization [17] is the impact measurement most mentioned in international quantitative studies [8,17-19]. Only Amara et al. [20] used the typology approach, and this study seems our only comparison option. However, because of our concentration on the details of health policy making in the Dutch local context, our research results are moderately comparable (for the outcome, for the dependent variables, and even for the associated variables). The different operationalization of the associated factors is especially problematic. This brings us to another issue - the need for the validation of instruments. It should be possible to reach international consensus on how research utilization should be measured, but further elaboration of these concepts is necessary. This could be achieved using, for example, the method of concept mapping by various international experts on research utilization. Consensus is also needed on presumed associated factors. However we acknowledge that it would be more difficult to reach international agreement on this because of the differences in policy context and processes. We also question how precise the measurements can be. For example, Ouimet et al. suggest that interaction activities can best be measured on an absolute scale [18]. In our earlier municipal case studies we found that it is sometimes difficult for people to remember this issue is because of the long term ongoing development of both research and policy [5].

As we described earlier, the regression models developed fit a specific policy context, and do not cover the dynamics of the entire policy network and policy process. However, if certain explanations of research utilization that are found in qualitative studies, for example [28-33], are true, we believe that other methodological approaches will provide additional information and parts of the puzzle. Quantitative studies are necessary to underpin qualitative findings and to underline the importance of the possible implications.

### Reflections on the conceptual framework

There are many conceptual frameworks circulating in the international scientific area of research utilization, of which our conceptual framework is one [9-12,18]. On some issues, our framework overlaps with others. For example, the independent variables of Landry et al. [34] and Amara et al. [20] relating to adaptation of the products (publications) mention issues (comprehension, credibility of the source, capacity to verify the quality of the results, appeal of the reports) that can be found in our list of barriers. But there is also overlap with Gourmet's model [18] where social interaction corresponds with our interaction questions, and where recognition of the values corresponds with our barriers.

In this study it turns out that interaction enhances research use. However we limited ourselves to direct ways of interactions between epidemiologists and local health officials. Taking the literature on nexus theories [13] and collaborative research [35] more advanced measures are possible of interaction. This will be challenge for future studies.

One important feature of our framework did not work out well; the classification of the barriers into four domains. The interrelations between these barriers could have multiple reasons. First of all, it is possible that, in the empirical setting, from the perspective of local officials, the meanings of the theoretical notions are hard to distinguish in practice. On the other hand, the way we operationalized the barriers and the sequence in which we questioned the respondents, could have influenced their answers. Because of these methodological shortcomings it is not possible to draw conclusions on the ways interaction between epidemiologists and local health officials are associated to the barriers, for example if the interactions influence the belief systems of officials. It is interesting to explore this clue in future studies because it could provide an explanation what interactions actually do on the interface between research and policy.

This study was limited to local health officials. According to our conceptual framework, there are many more stakeholders in the local policy process who could possibly use the epidemiological reports. This study provides no answer to this issue, so it becomes interesting to gain insight into these other groups in order to study research use in a whole policy network. However, to do this in a quantitative way will cost considerable research effort if it is to achieve a sufficient number of respondents.

### Concluding remarks

This study shows that conceptual use is more common among Dutch local health officials than other types of use. Probably this is precisely why the concept of evidence based policy, which, on many occasions, suggests instrumental use, should be replaced by evidence informed policy, which is related to conceptual use. Conceptual use itself was associated with a presentation given by the epidemiologist during the policy process, the presence of obstructions regarding the eots accessibility, and the local official's personal belief systems and interests. Furthermore, the results show that instrumental and symbolic use increased with the involvement of local officials in the research process.

The outcomes suggest that RPHS epidemiologists can use different strategies to improve research utilization. However, they do have to ask themselves beforehand what type of research utilization they want to achieve - should it be instrumental, conceptual, or symbolic. Either way, 'blurring the boundaries', and the enhancement of interfaces between epidemiologists and local health officials, like direct interactions into each other's work processes, is expected to create better possibilities for optimizing research use.

## Competing interests

The authors declare that they have no competing interests.

## Authors' contributions

JDG has drafted the manuscript and designed the study, acquired, analyzed, and interpreted the data. MVB and JM have been involved in analyzing and interpreting the data, drafting the manuscript, and revising it critically for important intellectual content. KP and HVO have revised the manuscript critically for important intellectual content and all authors have given final approval of the version to be published.

## Supplementary Material

Additional file 1 **The interrelations between the associated variables**. Click here for file

Additional file 2 **The combination for a new associated interaction variable for the regression analyses of conceptual use**. Click here for file

Additional file 3 **The additional ordinal regression analyses for instrumental use**. Click here for file

## References

[B1] Nutley SM Walter I Davies HTO Using Evidence. How research can inform public services 2007 Bristol, UK.: The Policy Press, University of Bristol 33

[B2] Nutley SM Walter I Davies HTO Using Evidence. How research can inform public services 2007 Bristol, UK.: The Policy Press, University of Bristol 232

[B3] Sackett DL Rosenberg WMC Muir Gray JA Brian Haynes RB Scott Richardson W Evidence-based medicine, what it is and what it isn't (Editorial) BMJ 1996 312 71 72 10.1136/bmj.312.7023.71 8555924 PMC2349778

[B4] Oxman AD Vandvik PO Lavis JN Fretheim A Lewin S SUPPORT Tools for evidence-informed health Policymaking (STP) 2: Improving how your organisation supports the use of research evidence to inform policymaking Health Res Policy Sys 2009 7 Suppl 1 S2 10.1186/1478-4505-7-S1-S2 PMC3271829 20018109

[B5] De Goede J Putters K van Oers HAM Utilization of epidemiological research during the development of local public health policy in the Netherlands: a Case Study Approach Social Science & Medicine 2012 74 707 714 10.1016/j.socscimed.2011.11.014 22265086

[B6] De Goede J Putters K van der Grinten T van Oers HAM Knowledge in process? Exploring barriers between epidemiological research and local health policy development Health Res Policy Sys 2010 8 26 10.1186/1478-4505-8-26 PMC2954864 20846419

[B7] Rich RF Oh CH Rationality and use of information in policy decisions: a search for alternatives Science Communication 2000 22 2 173 211 10.1177/1075547000022002004

[B8] Kothari A Birch S Charles C "Interaction" and Research Utilization in Health Policies and Programs: Does it Work? Health Policy 2005 71 117 125 10.1016/j.healthpol.2004.03.010 15563998

[B9] Hanney SR Gonzalez-Block MA Buxton MJ Kogan M The utilization of health research in policy-making: concepts, examples and methods of assessment Health Res Policy Syst 2003 1 2 28 10.1186/1478-4505-1-2 PMC151555 12646071

[B10] Innvaer S Vist G Trommald M Oxman A Health policy-makers' perceptions of their use of evidence: a systematic review J Health Services Res Policy 2002 7 4 239 244 10.1258/135581902320432778 12425783

[B11] De Leeuw E McNess A Crisp B Stagnitti K Theoretical reflections on the nexus between research, policy and practice Crit Publ Health 2008 18 1 5 20 10.1080/09581590801949924

[B12] Dobrow MJ Goel V Upshur REG Evidence-based Health Policy: Context and Utilization Soc Sci Med 2004 58 207 217 10.1016/S0277-9536(03)00166-7 14572932

[B13] Lavis J Davies H Gruen R Walshe K Farquhar C Working Within and Beyond the Cochrane Collaboration to Make Systematic Reviews More Useful to Healthcare Managers and Policy Makers Healthcare Policy 2005 1 2 21 33 19305650 PMC2585325

[B14] Mitton C Adair CE Mckenzie E Patten SB Perry BW Knowledge Transfer and Exchange: Review and Synthesis of the Literature Milbank Quarterly 2007 85 4 729 768 10.1111/j.1468-0009.2007.00506.x 18070335 PMC2690353

[B15] Jansen MWJ De Vries NK Kok G Van Oers HAM Collaboration between practice, policy and research in local public health in the Netherlands Health Policy 2008 86 2 295 307 10.1016/j.healthpol.2007.11.005 18178285

[B16] De Goede J Steenkamer B Treurniet H Putters K Van Oers HAM Public health knowledge utilization by policy actors: an evaluation study in Midden-Holland, The Netherlands Evidence and Policy 2011 7 1 7 24 10.1332/174426411X552972

[B17] Knott J Wildavsky A If dissemination is the solution, what is the problem? Knowledge: Creation Diffus Utilization 1980 1 4 537 578

[B18] Ouimet M Landry R Ziam S Bédard P The absorption of research knowledge by public civil servants Evidence & Policy 2009 5 4 331 35022470931

[B19] Belkhodja O Amara N Landy R Ouimet M The Extent and Organizational Determinants of Research Utilization in Canadian Health Services Organizations Science Communication 2007 28 3 377 417 10.1177/1075547006298486

[B20] Amara N Ouimet M Landry R New Evidence on Instrumental, Conceptual and Symbolic Utilization of University Research in Government Agencies Sci Commun 2004 26 1 75 106 10.1177/1075547004267491

[B21] Carifio J Perla R Ten Common Misunderstandings, Misconceptions, Persistent Myths and Urban Legends about Likert Scales and Likert Response Formats and their Antidotes J Social Sci 2007 2 106 116

[B22] De Heus P Van der Leeden R Gazendam B Applied data analyses. Techniques for non-experimental research in social sciences 1995 Utrecht: Lemma in Dutch

[B23] Allison PD Multiple regression 1999 CA Pine Forge Press: A primer Thousand Oaks

[B24] Evers A Lucassen W Meijer R Sijtsma K COTAN Grading the quality of tests. Dutch Institute for Dutch psychologists 2010 33 in Dutch

[B25] Schmidt M Joosen I Kunst AE Klazinga NS Stronks K Generating Political Priority to Tackle Health Disparities: A Case Study in the Dutch City of The Hague AJPH 2010 100 S1 10.2105/AJPH.2009.168526 PMC2837449 20147684

[B26] Van Dijk JP Local Health policy, scope and purposes PHD Thesis 2001 University of Groningen, The Netherlands in Dutch

[B27] Jamieson S Likert scales: how to (ab)use them Medical Educ 2004 38 1212 1218 10.1111/j.1365-2929.2004.02038.x 15566531

[B28] Bowen S Zwi AB Sainsbury P Whitehead M Killer facts, politics and other influences: what evidence triggered early childhood intervention policies in Australia? Evidence & Policy 2009 5 1 5 3222470931

[B29] Kothari A MacLean L Edwards N Increasing capacity for knowledge translation: understanding how some researchers engage policy makers Evidence & Policy 2009 5 1 33 5122470931

[B30] Behague D Tawiah C Rosato M Some T Morrison J Evidence-based policy-making: The implications of globally-applicable research for context-specific problem-solving in developing countries Social Sci & Med 2009 69 1539 1546 10.1016/j.socscimed.2009.08.006 19781839

[B31] Woelk G Daniels K Cliff j Lewin S Sevene E Fernandes B Mariano A Matinhure S Oxman AD Lavis JN Lundborg Stålsby Woelk G Daniels K Cliff J Lewin S Sevene E Fernandes B Mariano A Matinhure S Oxman AD Lavis JN Stålsby Lundborg C Translating research into policy: lessons learned from eclampsia treatment and malaria control in three southern African countries Health Res Policy Syst 2009 7 31 10.1186/1478-4505-7-31 20042117 PMC2809043

[B32] Jung T Nutley SM Evidence and policy networks: the UK debate about sex offender community notification Evidence & Policy 2008 4 2 187 20722470931

[B33] De Leeuw E McNess A Stagnitti K Crisp B. ts research, Jim, but not as we know it. Acting at the Nexus. Integration of research, policy and practice Final report 2007 VicHealth/Deakin University, Melbourne Australia

[B34] Landry R Lamari M Amara N Extent and Determinants of Utilization of University Research in Government Agencies Publ Administration Rev 2003 63 2 191 204

[B35] Lomas J Using 'linkage and exchange' to move research into policy at a Canadian foundation Health Affairs 2000 19 3 236 240 10.1377/hlthaff.19.3.236 10812803

